# Early Pregnancy Maternal Hepatocyte Growth Factor and Risk of Gestational Diabetes

**DOI:** 10.9734/BJMMR/2015/18632

**Published:** 2015-06-11

**Authors:** Michal Dishi, Karin Hevner, Chunfang Qiu, Neway G. Fida, Dejene F. Abetew, Michelle A. Williams, Daniel A. Enquobahrie

**Affiliations:** 1 Center for Perinatal Studies, Swedish Medical Center, Seattle, WA, USA; 2 Department of Epidemiology, University of Washington, Seattle, WA, USA; 3 Department of Epidemiology, Harvard School of Public Health, Boston, MA, USA

**Keywords:** Hepatocyte growth factor, gestational diabetes mellitus, obesity, physical activity

## Abstract

**Aims:**

We investigated associations of serum hepatocyte growth factor (HGF) with risk of gestational diabetes mellitus (GDM). We also examined whether pre-pregnancy overweight/obesity status or leisure-time physical activity (LTPA) modify these associations.

**Methods:**

In a nested case-control study (173 GDM cases and 187 controls) among participants of a pregnancy cohort, early pregnancy (16 weeks of gestation, on average) serum HGF was measured using enzyme-linked immunoassay. GDM was diagnosed using American Diabetes Association guidelines. Logistic regression was used to calculate odd ratios (ORs) and 95% confidence intervals (CI). Effect modifications by pre-pregnancy overweight/obesity status or LTPA during pregnancy were examined using stratified analyses and interaction terms.

**Results:**

Overall, we did not find significant associations of serum HGF with GDM risk (p-value> 0.05). However, compared with women who had low serum HGF concentrations (<2.29 ng/ml), women with high serum HGF concentrations (≥ 2.29 ng/ml) had 3.8-fold (95%CI: 1.30–10.98) and 4.5-fold (95%CI: 1.28–15.80) higher GDM risk among women who were overweight/obese, pre-pregnancy (body mass index≥25 kg/m^2^), or did not report LTPA, respectively. These associations were not present among women who were not overweight/obese (interaction p=0.05) or reported LTPA (interaction p=0.05).

**Conclusion:**

Overweight/obesity status and LTPA may modify associations of early pregnancy serum HGF with subsequent GDM risk.

## 1. INTRODUCTION

Gestational diabetes mellitus (GDM), a pregnancy-related disorder of glucose metabolism, complicates between 5–7% of all pregnancies [1–3]. GDM is associated with adverse fetal outcomes such as macrosomia and birth trauma. In addition, women with GDM represent a group of pregnant women with significant risk for developing glucose intolerance later in life [4,5]. Insulin resistance and reduced insulin secretion are known to be the central disturbances in the pathogenesis of GDM, similar to Type 2 diabetes. A variety of predisposing factors have been associated with GDM risk (including advanced maternal age, pre-pregnancy overweight and obesity status, and reduced physical activity) [6–11]. However, a significant gap remains in our understanding of risk factors and mediating mechanisms that contribute to GDM. Importantly, few prospective studies evaluated biomarkers that are associated with subsequent risk of GDM, limiting opportunities for prevention and early identification of women with higher risk for GDM.

Hepatocyte growth factor (HGF) is a mesenchymal-derived pleiotropic factor that regulates cellular growth and morphogenesis, as well as tissue regeneration and repair [12,13]. In experimental studies, investigators demonstrated that HGF plays a key role in pancreatic islet cell mass increase (and hyperinsulinemia), and insulin signaling in the liver [14]. Disruption of HGF and its receptor signaling enhanced pancreatic beta-cell death, and accelerated onset of Type 2 diabetes [15]. In humans, higher serum HGF concentrations were associated with both newly diagnosed and long-standing Type 1 diabetes [13], insulin resistance, and prevalent Type 2 diabetes [16]. The only previous study to examine relationships between HGF and GDM was an experimental study that reported that HGF/c-Met signaling is essential for maternal beta-cell adaptation during pregnancy and its absence/attenuation leads to GDM [17]. However, to our knowledge, association of circulating HGF with GDM has not been investigated in humans. Based on available literature, we hypothesized that HGF concentrations are positively associated with risk of GDM, and, that these associations will be stronger among women who also have additional risk factors of abnormal glucose metabolism (such as GDM) including obesity [18] and physical activity [19] that have been related to HGF.

Therefore, we investigated associations of early pregnancy HGF concentration with risk of GDM in a pregnancy cohort. We also examined whether these associations are modified by pre-pregnancy overweight/obesity status or leisure-time physical activity (LTPA) during pregnancy.

## 2. METHODS

The study population for this nested case - control study was drawn from participants of the Omega study. The Omega study is a prospective cohort study designed to examine maternal dietary and lifestyle risk factors of preeclampsia, GDM and other adverse pregnancy outcomes [20,21]. The study population comprised of women attending prenatal care clinics affiliated with Swedish Medical Center and Tacoma General Hospital, in Seattle, WA and Tacoma, WA, respectively. Women eligible for inclusion into the study were those who initiated prenatal care prior to 16 week of gestation. Women were ineligible if they were younger than 18 years of age, did not speak and read English, did not plan to carry the pregnancy to term, or did not plan to deliver at either of the two research hospitals. The procedures used in this study were approved by the Institutional Review Boards of the Swedish Medical Center and Tacoma General Hospital, WA. All participants provided written informed consent.

Participants were invited to participate in an in-person interview and provided blood and urine samples at or shortly after enrollment [16 weeks of gestation, on average). After delivery, maternal and infant records were reviewed for information on course and outcomes of pregnancy. From structured questionnaires and medical records, we obtained information on maternal socio-demographic characteristics, life style habits (such as leisure-time physical activity during pregnancy), family medical history, and reproductive and medical histories. Body mass index (BMI) was calculated as weight (in kilograms) divided by height (in meters) squared. Pre-pregnancy overweight/obesity status was defined as pre-pregnancy BMI ≥25 kg/m2.

GDM cases and controls were selected from pregnant women who enrolled in the Omega Study between 1996 and 2006. During this period, 5,063 eligible women were approached and 4,000 (approximately 79%) agreed to participate. A total of 3,886 pregnant women provided blood samples and completed interviews. The diagnosis of GDM was made using the American Diabetes Association 2004 guidelines [1]. In our study settings, all attendants of perinatal care clinics are screened at 24–28 weeks gestation using a 1-hour oral glucose load (50 g) screening test. Women who have post- load glucose concentrations >140 mg/dl were then followed-up within 1–2 weeks with a 3-hour oral glucose (100 g) tolerance test. Women were diagnosed with GDM if two or more of the four diagnostic glucose concentration measurements met or exceeded the following: fasting ≥95 mg/dl; 1 hour post-challenge ≥180 mg/dl; 2 hour post-challenge ≥155 mg/dl; and, 3 hour post-challenge ≥140 mg /dl. Among Omega study participants, we identified and sampled all 191 women who developed GDM. We also randomly sampled 191 control women who did not develop GDM. None of the GDM cases or selected controls had pre-gestational diabetes mellitus. There were 18 GDM cases and 4 controls that did not have adequate serum samples for serum HGF measurements. The final analytic population included 173 GDM cases and 187 controls.

Maternal serum HGF in early pregnancy (16 week of gestation, on average) was determined using commercially available HGF enzyme-linked immunoassay (Quantikine Human HGF ELISA R&D Systems Cat# DHG00 Minneapolis, MN, USA) according to the manufacturer’s recommendations. All assays were performed by one technician who was blinded to the case control status of study participants. The Inter-and Intra-assay coefficients of variations for the measurements were <10%.

## 3. STATISTICAL ANALYSIS

We examined distribution of selected maternal characteristics among GDM cases and controls. Statistically significant differences between the groups were evaluated using Student t test p-values for continuous variables and chi square test p-values for categorical variables.

Unadjusted and adjusted logistic regression models were used to examine associations of serum HGF concentrations with risk of GDM. Adjusted models included *a priori* identified potential confounders and variables that altered unadjusted regression estimates by 10% or more. Final adjusted models included covariates for maternal age, race/ethnicity, family history of diabetes, pre-pregnancy BMI, and leisure-time physical activity during the pregnancy. Serum HGF was assessed both as a continuous and categorical (quartiles, using the lowest quartile as the referent) variable. Cut-offs for quartiles of early pregnancy serum HGF concentrations were determined based on HGF measurement values among controls. Regression models were used to compute unadjusted and adjusted odds ratios (ORs/aORs) and corresponding 95% confidence intervals (CIs). P-value for trend was computed by assessing linear trend across increasing quartiles of serum HGF concentrations.

We evaluated our hypotheses of interactions between serum HGF concentrations and pre-pregnancy overweight/obesity status or leisure-time physical activity (LTPA) during early pregnancy on GDM risk using stratified models and interaction terms as follows. In stratified analyses, we examined whether associations of high/low serum HGF concentrations (using the median, ≥2.29 ng/ml, as cut-off) with GDM risk differ among strata that were defined by either pre-pregnancy overweight/obesity (BMI≥25 kg/m2) status or LTPA (active/not active) status. We also assessed whether the joint associations of serum HGF concentrations and pre-pregnancy overweight/obesity status with risk of GDM was greater than expected given potential independent associations. For this analyses, we created a variable that categorized women as (1) HGF<2.29 ng/ml and BMI<25 kg/m2, (2) HGF≥2.29 ng/ml and BMI<25 kg/m2, (3) HGF<2.29 ng/ml and BMI≥25 kg/m2, or (4) HGF≥2.29 ng/ml and BMI≥25 kg/m2. An analogous variable was created to assess joint associations of serum HGF concentrations and LTPA with GDM risk. Statistical significance of interactions was determined by evaluating p-values of interaction terms between high/low serum HGF and either pre-pregnancy overweight/obesity status or LTPA (active/not active) status in respective multivariable logistic regression models.

All analyses were conducted using SPSS (version 18) statistical software. Statistical significance was defined as two-sided p< 0.05.

## 4. RESULTS

Selected study participant characteristics are shown in Table 1. GDM cases were older and had higher pre-pregnancy BMI compared with controls (mean age 34.2 vs. 33.0 years and pre-pregnancy BMI 26.6 vs. 23.4 kg/m^2^ for GDM cases and controls, respectively). GDM cases were also more likely to report a positive history of hypertension, family history of diabetes, or a family history of hypertension compared with controls (all p-values<0.05). Controls were more likely to be non-Hispanic White compared with GDM cases (86% vs. 70.5%, respectively). Mean serum HGF concentrations were 2.02 ng/ml and 1.95 ng/ml among GDM cases and controls, respectively (p-value=0.28).

Overall, we did not find significant associations of serum HGF concentrations with GDM risk when serum HGF was modeled as a continuous (p-value= 0.361) or categorical variable (quartile trend p-value=0.479) (Table 2). A statistically insignificant 1.2-fold increase in GDM risk was observed among women who were in the highest quartile for serum HGF when compared with women in the referent (lowest) quartile for serum HGF (Adj. OR: 1.22 95%CI: 0.65–2.28).

In stratified analyses, associations of serum HGF concentrations with GDM risk was observed among women who were overweight/obese, pre-pregnancy (Table 3). Compared with women who had low serum HGF concentrations (<2.29 ng/ml), women with high serum HGF concentrations (≥ 2.29 ng/ml) had 3.8-fold (95%CI: 1.30–10.98) higher GDM risk among women who were overweight/obese. Similar high serum HGF-related increase in GDM risk was not observed among non-overweight/obese women (Adj. OR: 1.12, 95%CI: 0.61–2.04). Women who had high serum HGF and were overweight/obese had a 7-fold (95%CI: 2.58–18.54) higher risk of GDM compared with women who had low serum HGF and were not overweight/obese (p-value for interaction=0.05).

Similarly, associations of serum HGF concentrations with GDM risk were observed among women who did not report LTPA during current pregnancy (Table 4). Compared with women who had low serum HGF concentrations (<2.29 ng/ml), women with high serum HGF concentrations (≥ 2.29 ng/ml) had statistically significant higher GDM risk among women who were inactive (Adj. OR:4.50, 95%CI:1.28–15.80) but not among women who were active (Adj. OR:1.17, 95%CI:0.66–2.06). Women who had high serum HGF and were inactive had a 2.7-fold (95%CI:1.04–6.94) higher risk of GDM compared with women who had low serum HGF concentrations and were active (interaction p-value 0.046).

## 5. DISCUSSION

In the current study, we did not find overall statistically significant associations between early pregnancy serum HGF concentrations and GDM risk. However, we found that pre-pregnancy overweight/obesity status and LTPA modify associations of early pregnancy HGF with subsequent GDM risk. Increased serum HGF related risk of subsequent GDM was observed only among women who overweight/obese during pre-pregnancy or did not report LTPA during the pregnancy (p-values for interactions 0.05 and 0.046, respectively).

Previous studies have reported potential associations of higher serum HGF concentrations and increased type 2 diabetes risk. For instance, in the study among post-menopausal women, participants in the highest tertile for serum HGF concentrations had a 2.4-fold (95% CI1.12–5.47) higher risk for prevalent diabetes compared with participants in the lowest tertile for serum HGF concentrations [16]. Satani et al. [22] reported that mean serum HGF concentrations of patients with type 2 diabetes mellitus was 895 pg/ml (standard deviation 408 pg/ml), a level considerably higher than the normal range (<265 pg/ml).

To our knowledge, the only study that investigated serum HGF concentrations and GDM risk was an experimental study conducted by Demirci et al [17]. In that study, researchers investigated the role of HGF in the maternal beta cell adaption during pregnancy in lab mice by knocking out the receptor of HGF, c-met, from islet cells. In the knock-out pregnant mice, beta cell mass was decreased and this was accompanied by increased blood glucose, decreased plasma insulin and impaired glucose tolerance, hallmarks of GDM [17].

These findings are in line with experimental investigations of Garacia-Ocana and his colleagues who found that HGF increases beta cell mass in vivo [23]. In another recent experimental study to investigate a possible cause-effect relationship between increase in circulating HGF levels and compensatory islet hyperplasia / hyperinsulinemia, a strong correlation between HGF and the compensatory mechanism was demonstrated in three animal models of insulin resistance [14]. HGF increased beta cell mass in a dose-dependent manner; blocking HGF shuts down the compensatory mechanisms; and increases in HGF levels preceded the compensatory response associated with insulin resistance [14].

Overall evidence from previous studies indicate that HGF level increases in metabolic syndrome [24] and that HGF plays a role in insulin resistance and the development of T2DM. However, in our study we found that associations of higher early pregnancy serum HGF concentrations with risk of GDM were limited to women who were overweight/obese, pre-pregnancy, or who did not report LTPA. Our findings highlight the potential importance of complex relationships of HGF levels with other GDM risk factors in human populations.

Several studies have reported associations of higher HGF levels with adiposity markers. Circulating levels of HGF were elevated (up to threefold) among obese individuals compared with lean individuals (p<0.0001) [18]. Since pre-pregnancy obesity is a risk factor to GDM, we can contemplate that associations of higher HGF levels with risk of GDM may be exaggerated in the setting of pre-pregnancy obesity, as observed in the current study. Whether HGF mediates associations of pre-pregnancy BMI with risk of GDM is a potential area of future investigations.

Associations of physical activity and inactivity during pregnancy on the risk for GDM have been reported in previous studies [10,25]. Physical activity, especially vigorous activity before pregnancy and at least light to moderate activity during pregnancy, may reduce risk for abnormal glucose tolerance and GDM [26]. In a meta-analysis that reviewed the current evidence on the relationship between physical activity and GDM risk, Tobias et al. [25] reported that higher levels of physical activity before pregnancy or in early pregnancy are associated with significantly lower risk of developing GDM. The mechanisms that increased physical activity may cause reduction in the risk of GDM are multifaceted. Research among non-pregnant women indicates exercise-induced physical activity related improvement in glycemic control [27] that involves increased insulin-mediated and noninsulin mediated glucose disposal [28]. Some of the non-insulin mediated glycemic control may be accounted for by decreased oxidative stress, decreased fat mass, and increases in muscle mass [27]. Other studies have also shown that exercise has anti-inflammatory effects that may improve insulin sensitivity [29,30]. In our study, associations of higher HGF concentration with increased GDM risk was observed only among women who reported no LTPA. One potential reason for this observation may be that women with no LTPA have a lower “reserve” in glucose control and are more likely to be affected by higher HGF levels. The relationships between HGF and physical activity are less clear and it is not known whether HGF potentially mediates associations of physical activity with risk of GDM.

Several strengths of our study deserve mention. To our knowledge, this is the first population study to investigate associations of HGF with risk of GDM. The prospective design of the study helps clarify temporal relationships. We used strict criteria for GDM diagnosis, minimizing misclassification. Our study population is a well-characterized pregnancy cohort and the extensive data collection allowed us to control for several potential confounders.

Several potential limitations should be considered when interpreting our results. Although we adjusted for several potential confounders, we cannot exclude the possibility of residual confounding or confounding by unmeasured variables. We used self-report LTPA to characterize habitual physical activity in early pregnancy which is prone to some misclassification. However, because of the prospective study design, misclassification is not conditional on pregnancy outcome (GDM status). We used a single measurement of early pregnancy HGF and thus cannot definitively comment on pre-pregnancy HGF levels. This would limit our assessment of the relationships between HGF and the modifying GDM risk factors we evaluated (pre-pregnancy obesity and LTPA). Previous studies have used both serum and plasma to measure HGF. Available evidence does not indicate potential differences in HGF-GDM associations dependent on whether HGF is measured in serum [13, 17,18] or plasma [14,16]. However, the relevance of our findings to plasma HGF measurements needs further exploration. Finally, generalizability of our findings may be limited because our study participants were predominantly non-Hispanic white and residents of the pacific northwestern region of the USA.

## 6. CONCLUSION

Pre-pregnancy overweight/obesity status and LTPA may modify associations of early pregnancy HGF with subsequent GDM risk. Our findings enhance understanding of GDM pathogenesis and relationships between risk factors in human populations. Further, our findings support consideration of early pregnancy serum HGF as a biomarker of subsequent GDM for preventative and diagnostic purposes, particularly among overweight/obese or physically inactive women. Future replication and mechanistic studies of observed interactions are warranted.

## Figures and Tables

**Table 1:** Selected characteristics of study participants according to gestational diabetes (GDM) case-control status

Characteristics	GDM (N=173)		Control (N=187)		P-value**
		
	n	%	n	%	
Maternal age (years)*	34.2±4.6		33.0±4.3		**0.01**
<35	89	51.4	117	62.6	
≥35	84	48.6	70	37.4	**0.03**
Non-hispanic white	122	70.5	161	86.1	**<0.003**
Maternal education< 12 years	8	4.6	5	2.7	0.59
Nulliparous	91	52.6	105	56.2	0.50
Family history of diabetes	53	30.6	28	15.0	**<0.001**
Smoking during pregnancy	13	7.5	9	4.8	0.35
Chronic Hypertension	18	10.4	7	3.7	**0.01**
No LTPA early in pregnancy	36	20.8	29	15.5	0.19
Pre-pregnancy BMI (kg/m2)	26.6±6.5		23.4±5.1		**<0.001**
<18.5	4	2.3	10	5.3	**<0.001**
18.5–24.9	84	48.6	129	69.0	
25.0–29.9	48	27.7	39	20.9	
≥30	37	21.4	9	4.8	
Serum HGF (ng/ml)	2.02±0.61		1.95±0.55		0.28

Abbreviation: LTPA: leisure time physical activity; BMI = Body Mass Index (kg/m^2^), HGF: Hepatocyte growth factor.

* Mean ± SD, otherwise number (%)

** P-values from Student’s T-test for continuous variables and Chi-square test for categorical variables

**Table 2:** Risk of gestational diabetes (GDM) according to maternal serum hepatocyte growth factor (HGF) concentrations in early pregnancy

Serum HGF (ng/ml) concentrations	GDM cases (N= 173)	Controls (N= 187)	Unadjusted OR (95%CI)	Adjusted OR (95%CI)*
Continuous	-------	-------	1.22 (0.85–1.74)	1.20 (0.81–1.77)
Categorical	N (%)	N (%)		
Quartile 1 (<1.6)	41 (23.7)	46 (24.6)	1.00 (referent)	1.00 (referent)
Quartile 2 (1.62–1.95)	37 (21.4)	46 (24.6)	0.90 (0.49–1.66)	0.72 (0.37–1.41)
Quartile 3 (1.96–2.28)	42 (24.3)	47 (25.1)	1.00 (0.55–1.81)	0.75 (0.39–1.43)
Quartile 4 (≥ 2.29)	53 (30.6)	48 (25.7)	1.24 (0.70–2.20)	1.22 (0.65–2.28)
P-value for trend			0.405	0.479

* Adjusted for maternal age, race/ethnicity, and family history of diabetes, pre-pregnancy BMI, and leisure time physical activity during the pregnancy

**Table 3:** Interactions of maternal serum hepatocyte growth factor (HGF) with pre-preganncy overweight/obesity status on risk of gestational diabetes (GDM)

High HGF concentrations & pre-pregnancy BMI*	GDM cases (N=173)	Controls (N= 187)	Stratified model OR (95%CI)	Joint model OR (95%CI)
			
	N (%)	N (%)		
HGF <2.29 ng/ml & <25 kg/m^2^	61 (35.5)	97 (51.9)	1.00 (referent)	1.00 (referent)
HGF ≥2.29 ng/ml & <25 kg/m^2^	27 (15.7)	42 (22.5)	1.12 (0.61–2.04)	1.08 (0.59–1.98)
HGF <2.29 ng/ml & ≥25 kg/m^2^	58 (33.7)	42 (22.5)	1.00 (referent)	1.96 (1.15–3.34)
HGF ≥2.29 ng/ml & ≥25 kg/m^2^	26 (15.1)	6 (3.2)	3.77 (1.30–10.98)	6.91 (2.58–18.54)
P for interaction term				0.050

* Pre-pregnancy overweight/obesity status defined as pre-pregnancy BMI≥25kg/m^2^.

All models adjusted for maternal race/ethnicity, family history of diabetes, and leisure time physical activity during pregnancy

**Table 4:** Interactions of maternal serum hepatocyte growth factor (HGF) with leisure-time physical activity (LTPA) during the pregnancy on risk of gestational diabetes (GDM)

High HGF concentrations & LTPA	GDM cases (N=173)	Controls (N= 187)	Stratified model OR (95%CI)	Joint model OR (95%CI)
			
	N (%)	N (%)		
HGF <2.29 ng/ml & active	101 (58.4)	118 (63.1)	1.00 (referent)	1.00 (referent)
HGF ≥2.29 ng/ml & active	36 (20.8)	40 (21.4)	1.17 (0.66–2.06)	1.16 (0.66–2.03)
HGF <2.29 ng/ml & inactive	19 (11.0)	21 (11.2)	1.00 (referent)	0.63 (0.29–1.36)
HGF ≥2.29 ng/ml & inactive	17 (9.8)	8 (4.3)	4.50 (1.28–15.80)	2.68 (1.04–6.94)
P for interaction term				0.046

All models adjusted for maternal race/ethnicity, family history of diabetes, and pre-pregnancy BMI

## ABBREVIATIONS

HGF: Hepatocyte growth factor
GDM: Gestational diabetes mellitus
LTPA: Leisure-time physical activity
CI: Confident interval
OR: Odd ratio
BMI: Body mass index
